# A Paradigm Shift in Renal Cell Carcinoma: Recognizing Hypoglycaemia as a Significant Paraneoplastic Syndrome

**DOI:** 10.7759/cureus.73276

**Published:** 2024-11-08

**Authors:** Madhumitha S, Deepankumar T, Krishnaswamy Madhavan, Anand C D, Janardanan Kumar

**Affiliations:** 1 Department of General Medicine, Sri Ramaswamy Memorial (SRM) Medical College Hospital and Research Centre, SRM Institute of Science and Technology, Chengalpattu, IND; 2 Department of Pathology, Sri Ramaswamy Memorial (SRM) Medical College Hospital and Research Centre, SRM Institute of Science and Technology, Chengalpattu, IND

**Keywords:** insulin like growth factor, non-islet cell tumor hypoglycemia, paraneoplastic syndrome, recurrent hypoglycaemia, renal cell carcinoma

## Abstract

Recurrent hypoglycaemia is a rare paraneoplastic syndrome associated with several cancers, including renal cell carcinoma (RCC). Here we present a curious case of a patient with type 2 diabetes mellitus (T2DM) who had numerous hospitalisations due to recurrent hypoglycaemia. A well-defined lesion at the lower pole of the right kidney and bilateral pyelonephritis were found during imaging investigations. A biopsy from the lesion confirmed clear cell carcinoma of the kidney and the most probable cause of recurrent hypoglycemia was paraneoplastic syndrome related to RCC. Analysis of Insulin-like growth factor levels revealed that recurrent hypoglycaemia might be the result of non-islet cell tumor-hypoglycaemia (NICTH). This case emphasizes how crucial it is to rule out paraneoplastic syndromes like NICTH in individuals presenting with hypoglycaemia that cannot be explained, even when pre-existing diabetes is present. Furthermore, this study emphasizes the need to include RCC in the differential diagnosis.

## Introduction

Hypoglycaemia is a common medical emergency among diabetic individuals receiving insulin or sulfonylureas [1]. It is linked to non-islet cell tumour hypoglycaemia (NICTH) and is occasionally reported. Research has shown that NICTH, a hypoglycaemia syndrome, can be associated with any type of tumour other than insulinoma [2]. It is a rare paraneoplastic syndrome in which the tumour secretes high molecular weight insulin-like growth factor (IGF-II), causing hypoglycaemia.

Recurrent hypoglycaemia can occur as a paraneoplastic syndrome in carcinomas, most commonly in hepatocellular carcinoma (HCC) and pancreatic adenocarcinoma [1-3]. In HCC, hypoglycaemia has been reported in 4-27% of patients and is associated with poor prognosis [1,3]. Pancreatic adenocarcinoma presenting with recurrent hypoglycaemia is an infrequent manifestation [2]. Other malignancies, such as breast cancer and squamous cell carcinoma of the lung, can also present with NICTH in rare cases [4,5]. In this case, recurrent hypoglycaemia occurs as a rare clinical manifestation of paraneoplastic syndrome related to renal cell carcinoma (RCC) in a patient with a history of type 2 diabetes mellitus (T2DM).

## Case presentation

A 65-year-old male patient presented to the casualty department with symptoms of burning micturition for three days, diffuse pain in the abdomen, and diminished responsiveness since the evening. His past medical history included T2DM for five years, Parkinson's disease (PD) for five years, coronary artery disease (CAD) with a history of percutaneous transluminal coronary angioplasty (PTCA) 10 years prior, and pulmonary tuberculosis (PTB) treated 20 years ago.

He experienced five hospital admissions between November 2022 and March 2024. During his first admission on November 12, 2022, he was admitted because of giddiness secondary to hypotension and was diagnosed with sepsis secondary to bilateral pyelonephritis accompanied by elevated blood sugar levels (capillary blood glucose (CBG) 373 mg/dL). He received treatment and was discharged with medications for T2DM, PD, and CAD. In June 2023, he was readmitted with complaints of giddiness secondary to benign paroxysmal positional vertigo (BPPV). Despite treatment with insulin and oral antidiabetic drugs, his blood sugar level remained uncontrolled (CBG 280 mg/dL). Fundoscopy revealed non-proliferative diabetic retinopathy. The patient was discharged after stabilization. His third admission occurred in September 2023, presenting again with giddiness persisting for four days. During this stay in the hospital, he experienced recurrent hypoglycaemia, prompting the discontinuation of oral hypoglycaemic agents (vildagliptin + metformin 50/500 mg twice daily) and advised for self-monitoring of CBG at home upon discharge. In January 2024, he returned to the Emergency Room because of decreased response, decreased appetite, and continued giddiness. His initial CBG level was critically low, around 24 mg/dL, necessitating treatment with 25% dextrose and continuous monitoring. Imaging studies revealed age-related cerebral atrophy on a computed tomography (CT) brain scan, bilateral renal cortical echoes, and cystitis on abdominal ultrasound. A subsequent CT abdomen scan revealed bilateral pyelonephritis with moderate hydronephrosis. A urine culture revealed *Klebsiella pneumoniae*, and antibiotic therapy was administered. Despite the cessation of oral hypoglycaemic agents for four months, the patient continued to experience recurrent hypoglycaemia and was discharged with instructions for home CBG monitoring and regular meals.

**Figure 1:**
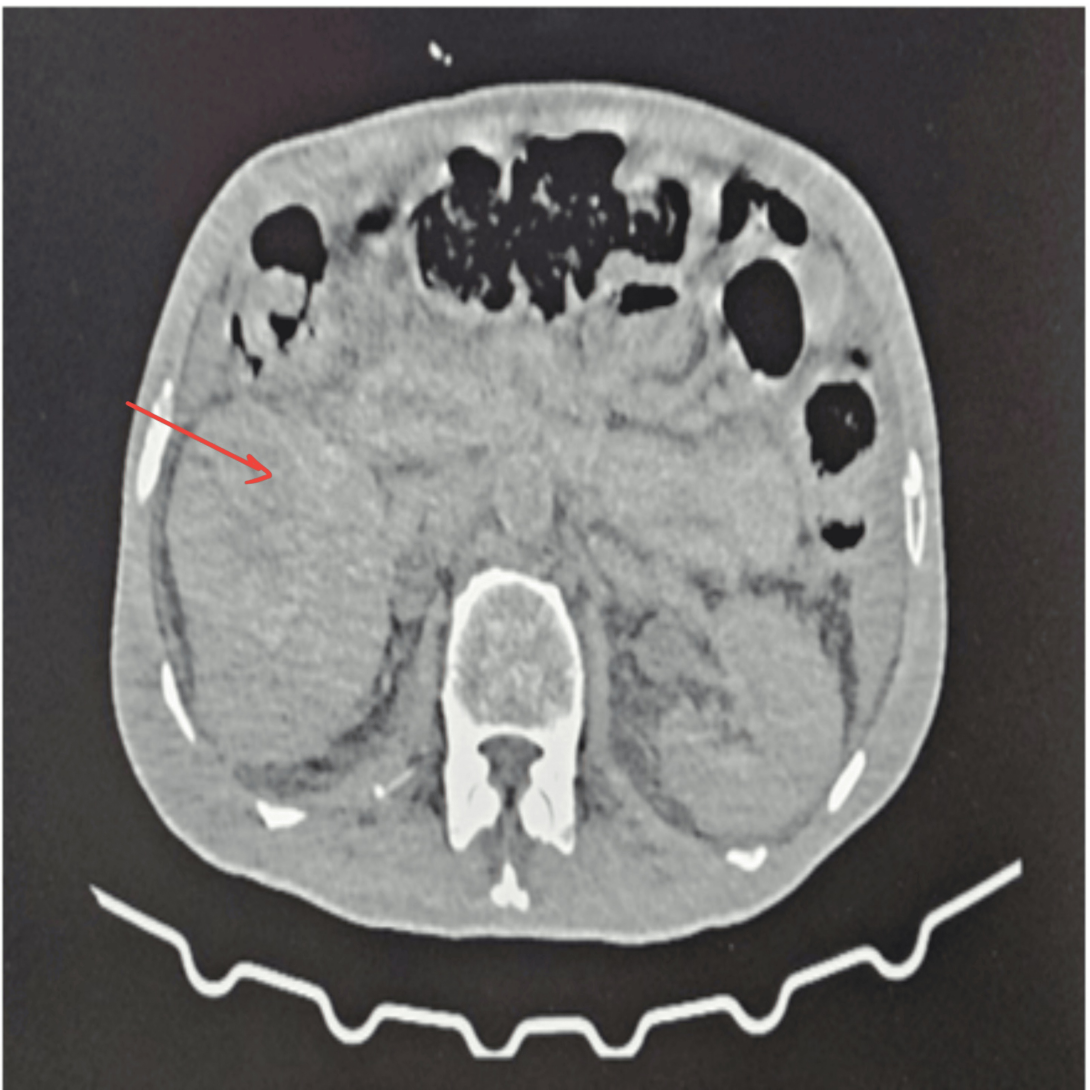
CT Abdomen showing enlarged right kidney measuring 14.9x6.7 cm (red arrow), multiple thick-walled hypodense collections in mid and lower poles of the right kidney (the largest measuring 7.3x4.1 cm) with minimal perinephric stranding. Right mild hydronephrosis is noted. The left kidney measures 11.4x4.6 cm with minimal perinephric fat stranding, which is suggestive of infective aetiology.

In March 2024, the current presentation, he was admitted with a decreased response, non-bilious vomiting (two to three episodes/day), diffuse abdominal pain lasting three days, and burning micturition for three days accompanied by chills and rigours. The patient also reported fatigue. Physical examination revealed drowsiness, pallor, and suprapubic tenderness. His vitals were stable. Initial CBG was low but improved to 109 mg/dL after administration of 25% dextrose. Blood samples were taken during the hypoglycaemia episodes for evaluation which revealed low normal fasting C-peptide and insulin levels, with normal serum cortisol levels. Abdominal CT findings revealed enlarged right kidney with minimal perinephric fat stranding and multiple thick-walled collections suggestive of renal abscess (Figure 1).

**Figure 2:**
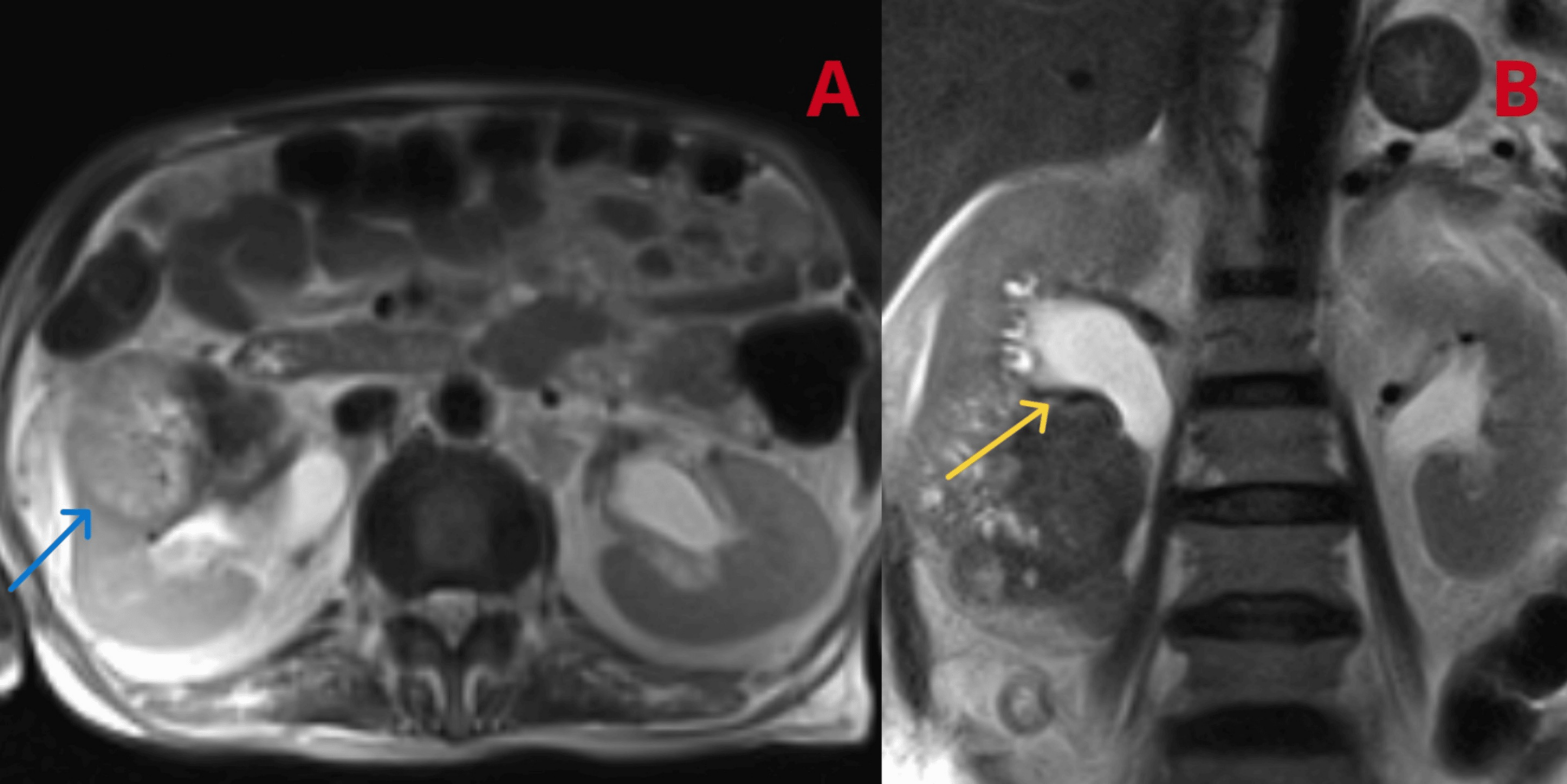
MRI abdomen and pelvis; (A) Right kidney appears enlarged and ill-defined heterogenous signal intensity lesion mid and lower pole measuring 9.6x7.1 cm (blue arrow), (B) Moderate hydroureteronephrosis noted in the right kidney (yellow arrow). The left kidney is normal in size and signal intensity. Pelvicalyceal system is not dilated.

Management included intravenous (IV) antibiotics for a suspected renal abscess, leading to admission to the intensive care unit (ICU) because of recurrent hypoglycaemic episodes necessitating hourly CBG monitoring and maintenance of IV dextrose. A Urology opinion was sought and CT-guided aspiration of renal abscess was suggested. CT-guided aspiration of the renal abscess yielded no fluid; hence, magnetic resonance imaging (MRI) of the abdomen and pelvis was done which showed an ill-defined heterogeneous lesion involving the mid and lower poles of the right kidney, raising suspicions of a renal mass or pyelonephritis with abscess formation (Figure 2).

**Figure 3:**
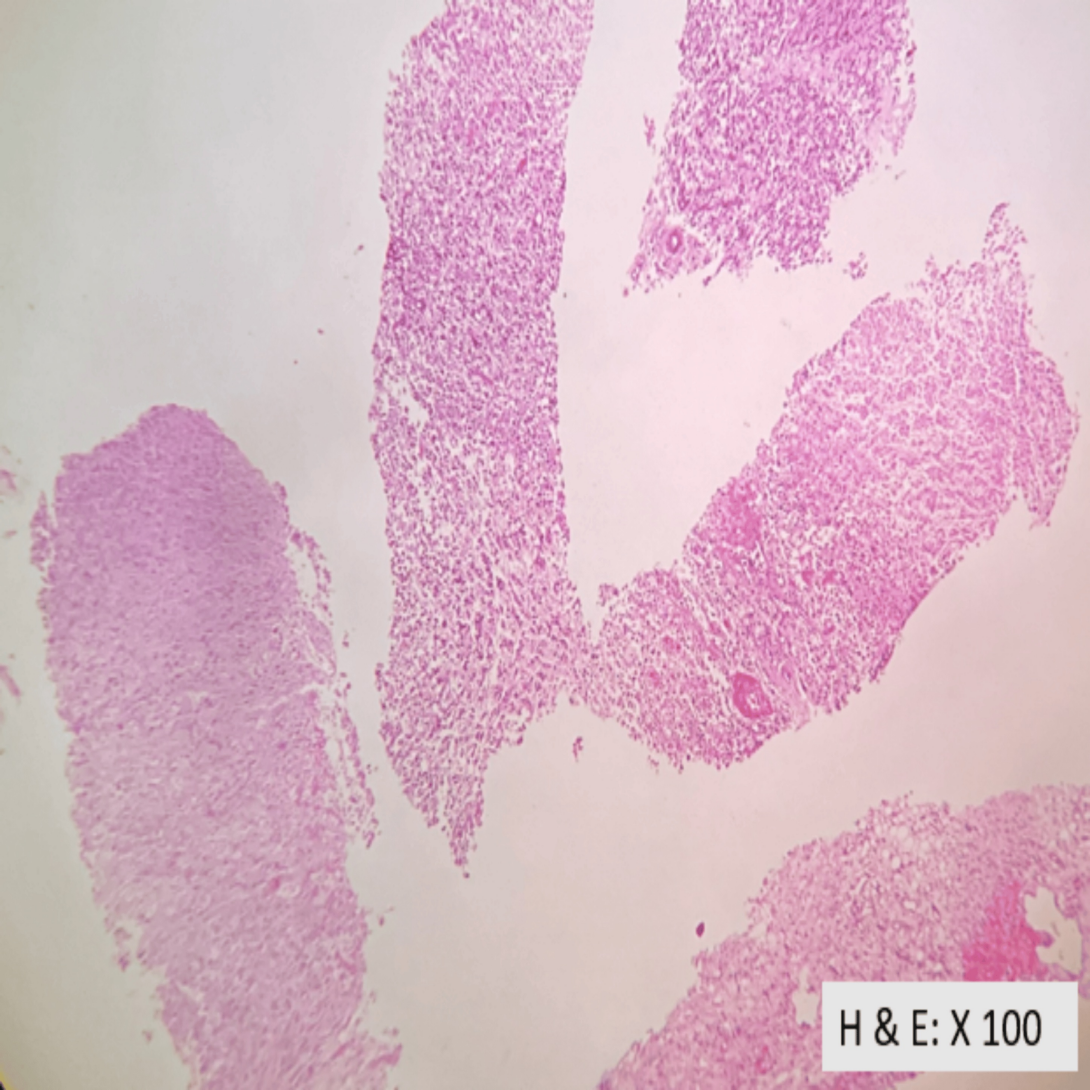
Core needle renal biopsy showing an infiltrating tumour arranged in sheets, alveolar pattern, and nest. Focal areas of necrosis is noted.

**Figure 4:**
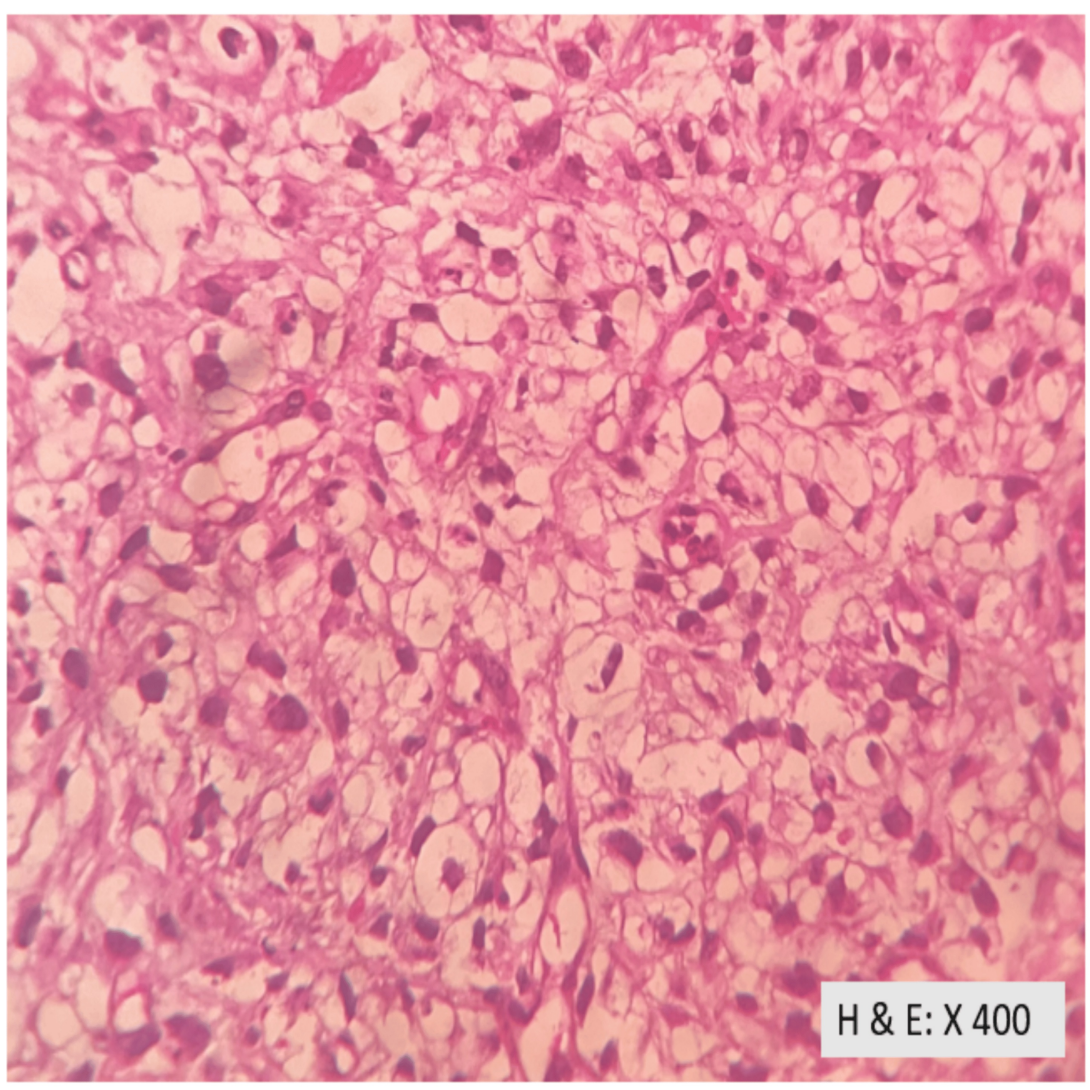
The tumour cells are round to polygonal with abundant clear vacuolated cytoplasm, some with eosinophilic cytoplasm, vesicular to hyperchromatic nuclei, moderate to marked nuclear pleomorphism, some with prominent nucleoli.

**Figure 5:**
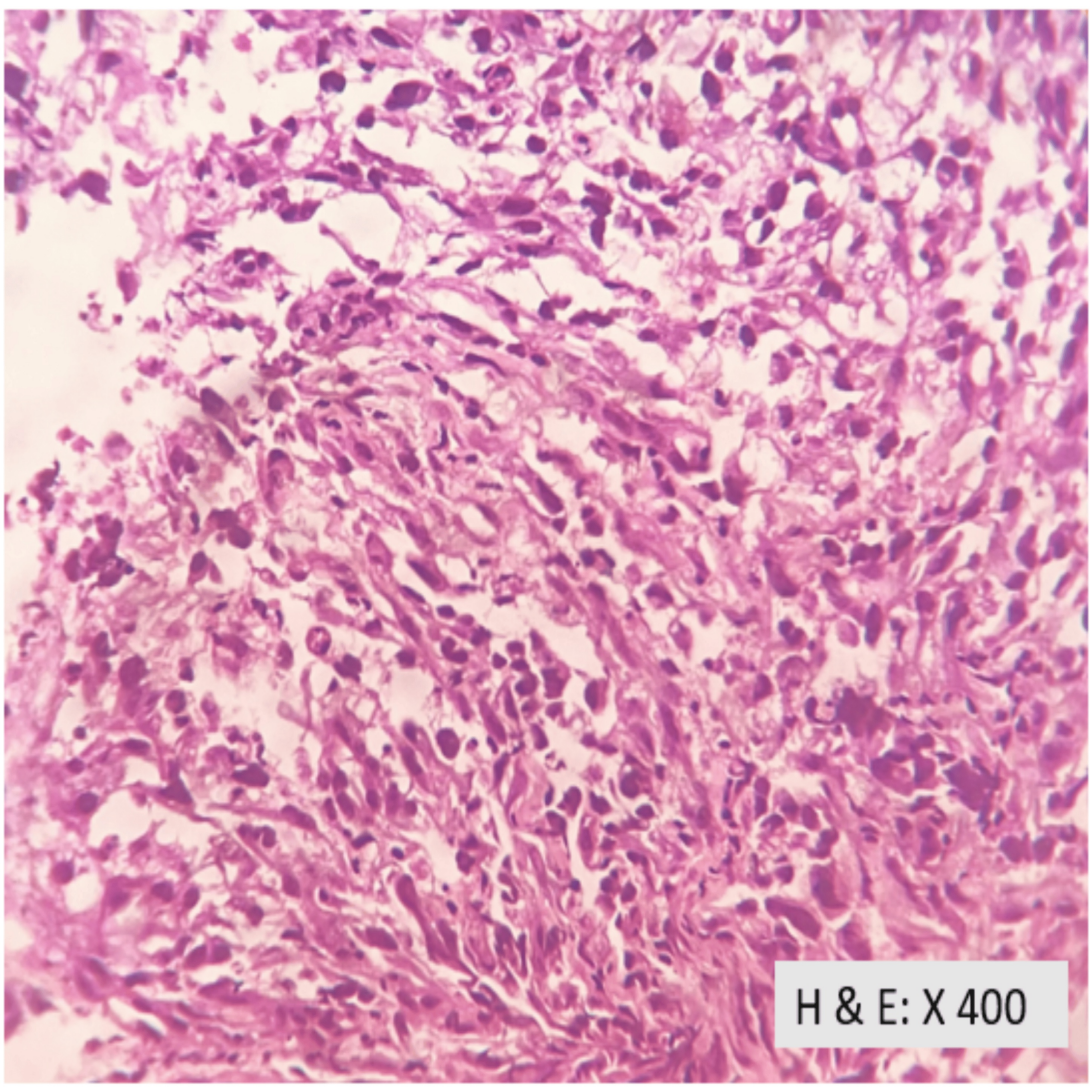
Focal areas of high-grade nuclear features with marked nuclear pleomorphism with oval to spindle-shaped hyperchromatic nuclei.

Ultrasound-guided biopsy confirmed clear cell carcinoma of the kidney (Figure 3-5).

Because renal abscesses and renal tumours can coexist, the high suspicion of an underlying renal tumour was increased when the CT-guided aspiration revealed no pus, which paved the way to proceed with renal biopsy and confirmed clear cell carcinoma of the kidney.

We proceeded with other investigations using the knowledge we had acquired from the literature to examine the connection between recurrent hypoglycaemia and RCC. IGF levels were sent in which IGF I level was 45 mcg/L (normal range 90-360 mcg/L) and IGF II level was 836 ng/mL (normal range 333-967 ng/mL) with IGF II/IGF I ratio more than 10. Hence, we concluded paraneoplastic syndrome from RCC was the likely cause of recurrent hypoglycaemia. Positron emission tomography-CT (PET-CT) confirmed the presence of a renal mass obstructing the ureter. The patient was placed on palliative treatment and there have been no further episodes since then.

## Discussion

RCC-induced paraneoplastic syndromes have been reported to occur in 10-40% of patients. Some of the more common paraneoplastic syndromes in RCC include hypercalcemia, anaemia, fever, cachexia, ectopic production of parathyroid hormone-related protein or erythropoietin and amyloidosis [6]. Hypoglycaemia is rarely listed as a possible paraneoplastic syndrome of RCC [6]. Tumours producing hypoglycaemia are classified into insulin-producing tumours and less common non-islet cell tumours [7]. Among many aetiologies of neoplasms that cause hypoglycaemia, insulinoma arising from pancreatic islet cells is the most recognised [6]. The differentiating features between NICTH and other causes are low levels of insulin in NICTH whereas levels of insulin are high in other causes. In NICTH, IGF-II level is high or normal and IGF-I level is low, whereas in other causes, both are in the normal range.

NICTH is defined as hypoglycaemia that is induced by a tumour other than insulinoma and IGF-II is the major cause of NICTH which can be diagnosed with elevated IGF-II levels, low or low normal insulin levels [7]. NICTH is most commonly associated with mesenchymal tumours such as fibrosarcoma, mesothelioma, and hemangiopericytoma. Epithelial tumours, particularly hepatocellular carcinoma, also often cause NICTH [8,9]. Other reported tumour types include adrenocortical carcinoma, stomach carcinoma, pancreatic carcinoma, medullary thyroid carcinoma, lymphoma/leukaemia and carcinoid syndrome. Additionally, NICTH can occur in RCC, although it is relatively rare. Tumours associated with NICTH are typically large, often exceeding 10 cm in diameter. Despite its rarity, NICTH is considered a paraneoplastic syndrome whose incidence may increase with rising malignancy rates [10-13].

Overproduction of the proprotein form of IGF-II, also known as "big IGF-II," is the main cause of NICTH. Big IGF-II has a higher molecular weight than mature IGF-II because of incomplete processing of the precursor protein. Structurally and functionally similar to insulin, big IGF-II can attach and activate insulin receptors, contributing to hypoglycaemia [14,15]. Tumours producing big IGF-II overwhelm the processing enzymes, leading to its accumulation and subsequent insulin-like effects, causing recurrent and severe hypoglycaemia through multiple mechanisms which include increased peripheral glucose uptake, reduced lipolysis, reduced free fatty acids, reduced hepatic gluconeogenesis, reduced glycogenolysis, and decreased growth hormone secretion from anterior pituitary [16]. The presence of high insulin and high C-peptide levels point toward insulinoma and insulinogenic drugs while laboratory findings in NICTH often include decreased levels of insulin, C-peptide, growth hormone, and IGF as well as increased IGF-II/IGF-I ratio [14,15]. One of the cases was encountered with NICTH which was caused by recurrent RCC and confirmed big IGF -II was associated with hypoglycaemia [17]. 

Whether a tumour is resectable or not determines how it is managed. In most cases, surgical resection is curative. Moderate to low doses of glucocorticoids combined with pasireotide and recombinant growth hormone are medical treatments that show promise in managing NICTH when it is incurable [6,18]. So a high level of suspicion is warranted in the diagnosis of NICTH, especially in patients with large solid tumours who exhibit recurrent fasting hypoglycaemia and non-specific symptoms [14,15].

## Conclusions

In this case, the manifestation of hypoglycaemia signalled a paraneoplastic syndrome, underscoring the importance of thorough investigation, even in patients with pre-existing diabetes. NICTH originating from RCC was diagnosed, emphasising the need to include RCC in the differential diagnosis of unexplained hypoglycaemia. The presence of high molecular weight IGF-II is a hallmark of NICTH which has to be checked if a patient has recurrent hypoglycemia. Imaging modalities, such as CT and MRI were instrumental in localizing the renal mass and ultrasound-guided renal biopsy paved the way for arriving at the final diagnosis. This case underscores the critical consideration of paraneoplastic syndromes with ambiguous clinical presentations.
